# A new regulatory mechanism for Raf kinase activation, retinoic acid-bound Crabp1

**DOI:** 10.1038/s41598-019-47354-7

**Published:** 2019-07-29

**Authors:** Sung Wook Park, Jennifer Nhieu, Shawna D. Persaud, Michelle C. Miller, Youlin Xia, Yi-Wei Lin, Yu-Lung Lin, Hiroyuki Kagechika, Kevin H. Mayo, Li-Na Wei

**Affiliations:** 1 Department of Pharmacology, University of Minnesota, Minneapolis, MN 55455 USA; 2 Department of Biochemistry, Molecular Biology & Biophysics, University of Minnesota, Minneapolis, MN 55455 USA; 3 Minnesota NMR Center, University of Minnesota, Twin Cities, Minneapolis, Minnesota 55455 USA; 4 Tokyo Medical and Dental University, Institute of Biomaterials and Bioengineering, Tokyo, Japan

**Keywords:** Hormones, Cell signalling

## Abstract

The rapidly accelerated fibrosarcoma (Raf) kinase is canonically activated by growth factors that regulate multiple cellular processes. In this kinase cascade Raf activation ultimately results in extracellular regulated kinase 1/2 (Erk1/2) activation, which requires Ras binding to the Ras binding domain (RBD) of Raf. We recently reported that all-trans retinoic acid (atRA) rapidly (within minutes) activates Erk1/2 to modulate cell cycle progression in stem cells, which is mediated by cellular retinoic acid binding protein 1 (Crabp1). But how atRA-bound Crabp1 regulated Erk1/2 activity remained unclear. We now report Raf kinase as the direct target of atRA-Crabp1. Molecularly, Crabp1 acts as a novel atRA-inducible scaffold protein for Raf/Mek/Erk in cells without growth factor stimulation. However, Crabp1 can also compete with Ras for direct interaction with the RBD of Raf, thereby negatively modulating growth factor-stimulated Raf activation, which can be enhanced by atRA binding to Crabp1. NMR heteronuclear single quantum coherence (HSQC) analyses reveal the 6-strand β-sheet face of Crabp1 as its Raf-interaction surface. We identify a new atRA-mimicking and Crabp1-selective compound, C3, that can also elicit such an activity. This study uncovers a new signal crosstalk between endocrine (atRA-Crabp1) and growth factor (Ras-Raf) pathways, providing evidence for atRA-Crabp1 as a novel modulator of cell growth. The study also suggests a new therapeutic strategy by employing Crabp1-selective compounds to dampen growth factor stimulation while circumventing RAR-mediated retinoid toxicity.

## Introduction

All-trans retinoic acid (RA), an active metabolite of vitamin A, regulates various physiological processes such as organ development, vision, cellular growth, differentiation, immune system regulation, and apoptosis, etc. These processes are mainly mediated by nuclear RA receptors (RARs), which are ligand-dependent transcription factors that regulate the expression of numerous target genes in various cell types^1–4^.

Recently, increasing attention has been directed towards non-canonical activity of atRA, independent of its ability to regulate gene expression via nuclear RARs. Other potential cytosolic activity of atRA has also been reported. For instance, RARs can sometimes be localized outside the nuclei, becoming cytosolic or on plasma membrane, to activate several kinases including p38 mitogen activated protein kinase (MAPK), PI3 kinase, and extracellular-signal-regulated kinase 1/2 (Erk1/2)^5–7^. We have reported that atRA can rapidly activate Erk1/2 activity^8,9^, and determined a physiological relevance for this “non-canonical” activity of atRA in cell cycle control of stem cells such as embryonic stem cells (ESCs) and neuron stem cells (NSCs)^8,10^. In ESCs, atRA can very rapidly (within minutes) activate Erk1/2 in an RAR- and membrane signal-independent manner^8,9^. By using gene-knockout systems, we have unambiguously determined that cellular retinoic acid binding protein 1 (Crabp1) mediates the non-canonical (non-genomic) activity of atRA in regulating Erk1/2, ultimately reflected on altered cell cycle control of ESCs^9^ and expansion of NSC pool in hippocampus that affects mouse learning behavior^10^. However, how Crabp1 mediates the non-canonical activity of atRA to modulate Erk1/2 activation remains entirely unclear.

Erk kinases participate in numerous biological processes and can be regulated by various signal inputs^11^. Classical (canonical) Erk signaling begins with stimulation on the cell surface by growth factors binding to receptor tyrosine kinases (e.g. epidermal growth factor receptor), leading to activation of Ras GTPase. The activated Ras (GTP-bound) binds to the Ras binding domain (RBD) of the rapidly accelerated fibrosarcoma (Raf) kinase which then undergoes a conformational change leading to its enzymatic activation^12^. Raf activation is the first critical step in the growth signaling cascade^13–15^. Activated Raf then phosphorylates MAPK-Erk kinase 1/2 (Mek1/2), and then finally Mek1/2 phosphorylates Erk1/2^13,14,16^. Erk/MAPK signal strength and duration can be regulated by multiple mechanisms including Ras and Raf mutations, interactions with scaffold proteins, and crosstalk with other signaling components such as cAMP-protein kinase A and Ca^2+^-calcineurin, etc^14,16,17^. However, it is unclear which component(s) in the Raf-Mek-Erk1/2 signaling cascade is/are targeted by Crabp1.

Here we report Raf kinase as the direct target of atRA-Crabp1 and determine the molecular mechanism of Crabp1’s action, which occurs through direct interaction with the RBD of Raf kinase. Using NMR, we describe the interaction surface on Crabp1 proposed to interact with the RBD of Raf. Finally, we identify a Crabp1-selective, atRA-mimicking compound which can also modulate Raf activation in a Crabp1-dependent manner. The physiological significance of this new finding is discussed in the context of signal cross-talk between the endocrine/vitamin A and growth factors, which is key to homeostatic control for cell growth and differentiation.

## Results

### Crabp1 sequentially forms complexes with components of the Raf-Mek-Erk signaling pathway to modulate Erk1/2 phosphorylation

**Figure 1:**
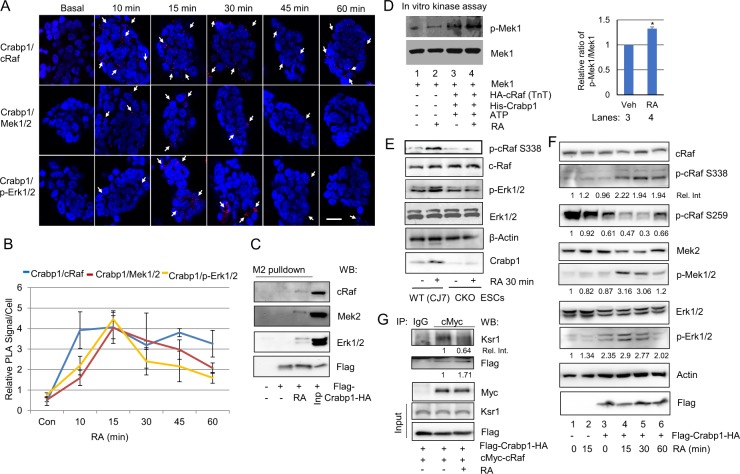
Crabp1 sequentially forms complexes with the components of Raf-Mek signaling pathway to modulate Erk1/2 phosphorylation. (**A**) PLA (Duolink) assays detect atRA-induced sequential formation of Crabp1, first with cRaf (top panel), then Mek1/2 (middle panel) and finally p-Erk1/2 (bottom panel). Nuclei are stained with DAPI. The red punctate represents complex formed with Crabp1. Representative microphotographs of various fields are shown, and scale bar refers to 20 µm. (**B**) Quantitation of PLA signals. The kinetics of Crabp1 forming complexes with the three key components in the MAPK pathway are plotted for relative numbers of PLA signal/cell each with a standard deviation. Experiments were performed three times. (**C**) Crabp1 is a scaffold protein for cRaf-Mek2-Erk1/2, and atRA induces scaffolding. HEK293T cells expressing Flag-Crabp1-HA were treated with 100 nM atRA for 15 min, and lysates were immunoprecipitated with M2 flag beads. WT cells were used as negative control. Inp refers to input control. (**D**) *Semi in vitro* kinase assay. Recombinant Mek1 is phosphorylated by TnT generated HA-cRaf and ATP in the absence or presence of His-Crabp1. Phosphorylation is increased by the addition of 100 nM atRA. Densitometric analyses western blot for lanes 3 and 4 are depicted at right (*P < 0.05, student t-test). (**E**) WT (CJ7) and CKO ESCs were treated with 100 nM atRA for 30 min, and relevant endogenous proteins in cell lysates were monitored. (**F**) atRA activates cRaf-Mek1/2-Erk1/2. WT and Flag-Crabp1-HA expressing HEK293T cells were treated with atRA for the indicated time, and relevant endogenous proteins in cell lysates were monitored via western blot. (**G**) Crabp1 competes with Ksr1. Lysates of HEK293T expressing Flag-Crabp1-HA and cMyc-cRaf were treated with atRA for 15 min followed by immunoprecipitation with anti-cMyc antibody and western blot analysis. Relative intensity is numerically marked.

We have previously reported membrane signal- and nuclear RAR-independent atRA–regulated Erk1/2 activation in a stem cell context, which requires Crabp1^8–10^. By screening known signaling components, we found that Crabp1 forms atRA-enhanced complexes with the Raf-Mek signaling components in ESCs. Figure 1A shows a Duolink proximity ligation assay for the real-time, sequential molecular scaffold formation of endogenous Crabp1 with the Raf-Mek-Erk signaling components, i.e., Crabp1-Raf/Mek/Erk in ESCs at different time points following atRA treatment (100 nM). The top panel shows that Crabp1 first associates with cRaf within 10 min of atRA treatment and the association sustains for 60 min. Subsequently, cRaf/Crabp1 forms complexes with Mek1/2 and p-Erk1/2 (the middle and lower panels, respectively). The kinetics of forming these Crabp1-containing molecular scaffolds indicate that these events occur sequentially (Fig. 1B), initiated as early as 10 min of atRA addition, and peaked at 15 min. Furthermore, co-immunoprecipitation demonstrates that Crabp1 forms stable complexes with cRaf, Mek2 and Erk1/2 in the presence of atRA (Fig. 1C, comparing lanes 2 and 3). These data suggest that Crabp1 may serve as a signal scaffold, or allosteric regulator, of cRaf, conveying Raf to Mek and Erk activation by forming an atRA-inducible signalosome activating the final target kinase, Erk1/2.

To determine whether Raf indeed is the immediate and direct target of atRA-Crabp1, we employed *in vitro* kinase assay for Raf using its direct substrate, Mek1. Figure 1D shows that Crabp1 is indeed involved in Raf-mediated Mek1 phosphorylation. In this *in vitro* kinase assay, atRA alone, without growth factor stimulation, has no effect on Raf activity (Mek1 phosphorylation; the 2^nd^ lane). In the presence of Crabp1, atRA can increase Raf activity (comparing the 3^rd^ and 4^th^ lanes). Thus, without growth factor input, atRA-Crabp1 alone is able to activate Raf and then Mek1. We further validated the requirement for Crabp1 in this non-canonical Raf/Erk activation in a physiologically relevant cellular context such as ESC. Figure 1E shows the comparison between wild-type (WT) and Crabp1-null (CKO)^9,10^ ESCs treated with atRA, without growth factor input, for 30 min. The data show that cRaf phosphorylation at S338 (p-S338), which is a critical residue for priming its full activation^13,16^, is elevated by atRA in the absence of growth factor only in WT ESCs (top, left), but not in CKO ESCs (top, right). Consistently, atRA-induced Erk1/2 phosphorylation is also detected only in WT ESCs, but not in CKO ESCs (top 3^rd^ panel). We then tested whether Crabp1, as a scaffold without growth factor input, can facilitate Raf-Mek-Erk activation by comparing Crabp1-negative and Crabp1-expressing HEK293T cells (Fig. 1F). As shown, atRA alone (without Crabp1, for 15 min) does not activate cRaf (p-S338), Mek1/2 and Erk1/2 (comparing lanes 1 and 2). Crabp1 alone (without atRA) also has little effect (lane 3). But atRA with Crabp1 (atRA-Crabp1) increases p-S338, peaking at 30 min (lane 5), and reduces p-S259, a mark of inactive cRaf, which seems to partially recover at a longer duration of treatment (60 min, the 2^nd^ panel; lanes 3–6). Consistently, phosphorylation of Mek1/2 and Erk1/2 peaks at 15 min of atRA treatment (lane 4), and tapers afterward (the 4^th^ and 6^th^ panels).

We then examined the relationship between Crabp1 and the well-known scaffolding protein kinase suppressor of Ras (Ksr1). Figure 1G shows that atRA reduces cRaf association with Ksr1, but increases cRaf association with Crabp1, suggesting competition between Ksr1 and Crabp1 for association with cRaf.

Together, these results show that Crabp1 is a new scaffold, or allosteric regulator, of Raf-Mek-Erk signaling complex. In the absence of growth factor stimulation, Crabp1 can mediate the non-canonical activity of atRA to activate Raf.

### atRA-Crabp1 dampens EGF-induced Erk1/2 activation

**Figure 2:**
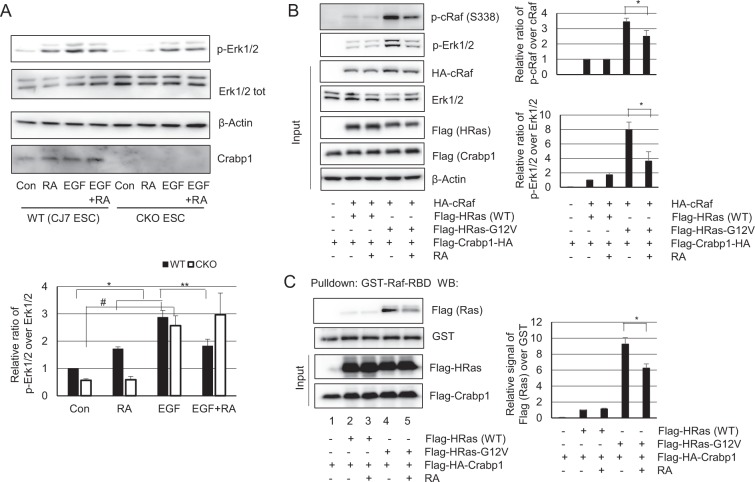
atRA/Crabp1 dampens canonical EGF and Ras mutation elicited Erk1/2 activation. (**A**) Dampening effects in ESCs. ESCs were pre-treated with atRA (100 nM, 30 min) and then with EGF (25 ng/ml, 5 min). Erk1/2 phosphorylation, the final signal of MAPK cascade, is shown (top panel). Blots are representatives of three independent experiments. Band intensity is statistically analyzed at bottom: *P < 0.01, ^#^P < 0.01 and **P < 0.05 (n = 4). (**B**,**C**) Dampening effects in Crabp1-positive HEK293T clones over-expressing other components as indicated, treated with, or without, atRA for 30 min. Anti-Flag antibody recognizes Flag-tagged Ras and Crabp1. (**B**) Phosphorylation of cRaf (at S338) and Erk1/2 was dampened by atRA in cells harboring the constitutively active mutant HRas (G12V), *P < 0.05 (n = 4). (**C)** Ras interaction with Raf (indicating activated Ras) in cells, monitored using GST-Raf-RBD beads (Millipore Sigma) to pulldown cell lysates in (**B**). The active Ras bound to the RBD of Raf is monitored. RBD: Ras binding domain. *P < 0.05 (n = 3).

To examine how atRA-Crabp1 signaling impacts on the growth factor mediated MAPK signaling, we determined the effect of atRA-Crabp1 on the receptor tyrosine kinase (RTK)-activation of Erk, stimulated by epidermal growth factor (EGF), in WT and CKO ESCs. The data show that atRA treatment alone (without EGF stimulation) rapidly increases Erk1/2 phosphorylation only in WT ESCs, but not in CKO ESCs, at an intensity apparently lower than that of EGF stimulation (25 ng/ml, 5 minutes). Importantly, pre-treatment with atRA before EGF stimulation in ESCs results in dampened Erk1/2 phosphorylation, as compared to Erk1/2 activation induced by EGF alone (Fig. 2A). This result indicates that while atRA-Crabp1 by itself can activate Erk1/2 under the basal condition without growth factor stimulation, atRA-Crabp1 in fact can negatively modulate (dampen) canonical, growth factor-activated Erk1/2.

It is known that Ras activation by RTKs on membrane initiates Raf/Erk signaling pathway, and mutations of Ras isoforms such as G12V of HRas occur as constitutive activators in 20–30% of various tumors. Further, these Ras isoforms (H, K, and N) are highly (~82–90%) conserved^18–21^. To investigate the dampening mechanism of atRA/Crabp1 in a growth factor stimulated situation, we expressed a WT Ras, or its constitutively active oncogenic mutant G12V (locked in the active GTP bound form) to drive canonical Erk1/2 activation in HEK293T cells without the need for growth factor treatment. As shown in Fig. 2B, over-expressing cRaf and WT Ras (locked in its inactive form in the absence of growth factors) elicits only negligible basal cRaf and Erk1/2 activation as expected (the 2^nd^ lane). In this cRaf and wild type HRas over-expression condition, atRA treatment alone (30 minutes, without Crap1) does not affect Raf or Erk phosphorylation) (Fig. 2B, upper two panels). As predicted, expressing the oncogenic Ras-G12V, to mimic robust growth factor stimulation, very strongly activates cRaf and Erk1/2 even without growth factor stimulation. In this condition, atRA effectively dampens Raf/Erk activation (Fig. 2B, upper two panels, compare two right lanes). These results show that atRA/Crabp1 can dampen oncogenic Ras-induced Raf activation, suggesting that atRA/Crabp1 can be a negative modulator for growth factor-stimulated or oncogenic MAPK pathway,

Further, in this system, we examined any potential molecular interplay of Crabp1 with Ras in regulating Raf using GST-Raf-RBD beads (Fig. 2C). In negative controls (lanes 1–3) WT Ras fails to interact with the RBD of Raf as predicted. In the presence of Crabp1 but not atRA, the constitutively active Ras-G12V effectively interacts with Raf (lane 4). But with the addition of atRA to this reaction, the interaction of active Ras-G12V with Raf is apparently reduced (lane 5). These results indicate that atRA-Crabp1 can inhibit active Ras-induce Raf phosphorylation, implicating that atRA-Crabp1 may compete with the active Ras for Raf interaction.

### Crabp1 competes with Ras for direct interaction with Raf

**Figure 3:**
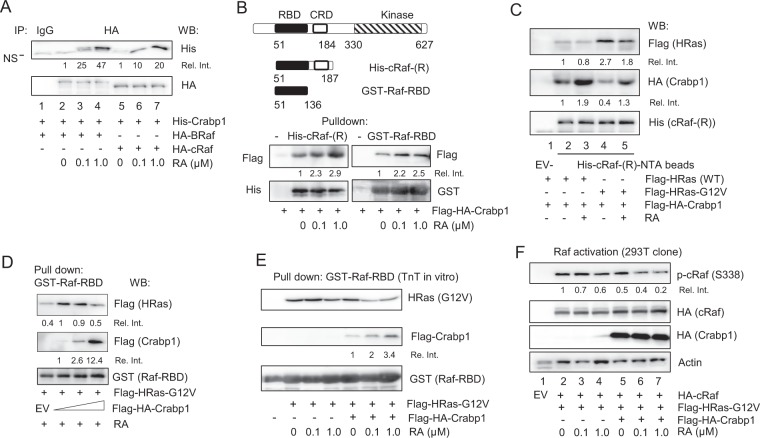
Crabp1 directly interacts with the RBD of Raf and competes with Ras for interaction with the RBD. (**A**) Direct protein interaction assay. TnT generated HA-BRaf or HA-cRaf, together with the purified His-Crabp1 was precipitated with anti-HA antibody, and its binding partner His-Crabp1 was detected by anti-His antibody. NS refers to a non-specific band. (**B**) Mapping the Crabp1-interacting domain of Raf. The constructs of regulatory domain (His-cRaf-(R)) and RBD (GST-Raf-RBD) (Cytoskeleton Inc) used for protein interaction assay are depicted (top). CRD: cysteine rich domain. Ni/NTA beads capturing His-cRaf-(R) or GST-Raf-RBD beads were used to pull down cell lysates containing Flag-HA-Crabp1. Anti-Flag antibody detects increased binding of Flag-Crabp1 to the RBD by RA (bottom). (**C**) Crabp1 competes with active Ras for Raf interaction. TnT-generated Flag-HRas (WT or G12V) and cell lysates containing Flag-HA-Crabp1 were pulled down by His-cRaf-(R) with or without atRA. Crabp1 was detected by anti-HA antibody, and Ras by anti-Flag antibody from the cut upper region. EV: empty vector. (**D**) Crabp1 dose-dependent effect in competing with the activated Ras. Increasing amount of Flag-HA-Crabp1 was mixed with the activated HRas (G12V) and pulled down with GST-Raf-RBD (Millipore Sigma) in the presence of atRA. Anti-Flag antibody detected HRas and Crabp1 on the same membrane. (**E**) atRA dose-dependent effect on Crabp1 competition with the active Ras. Active HRas (G12V) was incubated with increasing concentrations of atRA in the absence or presence of Flag-HA-Crabp1 and pulled down with GST-Raf-RBD beads (Cytoskeleton Inc). The pull-down products were detected by western blots with the indicated antibodies from the different membranes. (**F**) Direct assay of Raf activation (phosphorylation at S338) in cells provided with or without Crabp1. All blots are representatives of three independent experiments and relative fold intensity is numerically marked.

We thus extended our hypothesis that, Crabp1 may directly compete with the active Ras for Raf activation. We first determined the molecular basis of how atRA-Crabp1 intersected the canonical Raf signaling pathway using an *in vitro* protein interaction assay. Figure 3A shows that Crabp1 indeed directly interacts with Raf (both BRaf and cRaf tested here); further, the interaction is enhanced by atRA. We then used this type of *in vitro* direct protein interaction assay to dissect the interaction motif. Figure 3B shows that Crabp1 directly interacts with the regulatory domain of cRaf, particularly the RBD, which is also enhanced by atRA. As the RBD is highly conserved across all three Raf members (A, B and C)^22^, Crabp1 can potentially interact with all these Raf kinases.

Given that Crabp1 interacts with the highly conserved RBD of Raf, which is also the critical contact domain for Ras, it is tempting to speculate that atRA-Crabp1 may block canonical Raf activation by competing out growth factor activated Ras. We first validated that Crabp1 does not associate with Ras (data not shown). When both WT Ras (the inactive form) and Crabp1 are present (Fig. 3C, 2^nd^ lane), Raf preferentially interacts with Crabp1 (the middle panel) but not the inactive Ras (the top panel), and Raf interaction with Crabp1 is enhanced by atRA (lane 3). Importantly, when the oncogenic G12V mutant Ras (the active form) is used (the 4^th^ and 5^th^ lanes), Raf preferentially interacts with the active Ras (top panel) in the absence of atRA (the 4^th^ lane), but adding atRA to the reaction enables Crabp1 to effectively compete with the active Ras (the 5^th^ lane). By altering Crabp1 and atRA inputs, it is clear that atRA-Crabp1 competes with Ras-G12V in a Crabp1 (Fig. 3D) and atRA (Fig. 3E) dose-dependent manner. These *in vitro* studies clearly validate the competition of atRA-Crabp1 with the active Ras in forming complexes with Raf.

Finally, we examined the competition between atRA-Crabp1 and active Ras that underlies atRA’s ability to dampen Raf activation (phosphorylation) in cells using the reconstituted HEK293T cells and monitored Raf activation directly by its S338 phosphorylation. Figure 3F shows that, in the absence of Crabp1 (lanes 2–4), atRA has no effects on Ras-G12V-triggered Raf activation (no change in pS338 level, the top panel). However, in the presence of Crabp1 (lanes 5–7) atRA, dose-dependently, dampens Ras-G12V-triggered Raf activation (lowered pS338 levels). These in-cell results validate our conclusion that atRA-Crabp1 competes with active Ras for Raf activation, which underlines the dampening effect of atRA on growth factor-activated Raf-Erk signaling. The competition of Crabp1 with Ras is mediated by its direct interaction with the RBD of Raf, which is also the critical contact domain for Ras.

### Structural validation of Crabp1-Raf interaction

**Figure 4:**
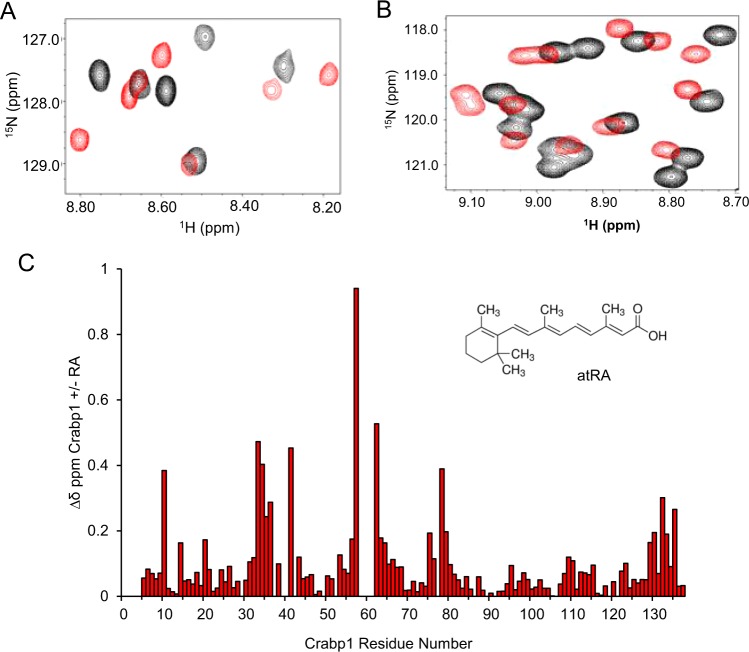
NMR analyses of atRA binding to Crabp1. (**A**,**B**) Expanded regions of ^1^H,^15^N HSQC spectra of 19 µM Crabp1 in the absence (black peaks) and presence of 21 µM atRA (red peaks), confirming atRA binding. (**C**) Maximal chemical shift changes observed between apo-Crabp1 and the atRA-Crabp1 bound complex. The atRA structure is shown in the inset. Chemical shifts were internally referenced to DSS (4,4-dimethyl-4-silapentane-1-sulfonic acid), and chemical shift differences were calculated as [(∆δ ^1^H)^2^ + (0.25 ∆δ ^15^N)^2^]^1/2^. NMR acquisition and sample conditions are described in the “Material and Methods”.

To evaluate the molecular feature of Crabp1 interaction with the RBD of Raf, we then employed NMR analyses to identify the interaction surface on Crabp1. Molecular and structural changes of Crabp1 have been examined in the past mainly with respect to atRA-induced conformational changes, especially the atRA-binding pocket. The molecular changes in its surface areas, as well as whether/how Crabp1 may interact with other molecules were never addressed. To substantiate our finding that Crabp1 can directly interact with the RBD of Raf, we thus employed NMR spectroscopy to determine the molecular features of Crabp1 complex formation with the RBD of Raf. Consistent with previous reports, our NMR studies demonstrate that Crabp1 binds atRA^23–27^. Figure 4A,B show ^15^N-^1^H HSQC spectral expansions of ^15^N-labeled Crabp1 in the absence of atRA (black peaks) overlaid with those of Crabp1 in the presence of atRA (red peaks). Interactions occur within the slow exchange regime on the chemical shift time scale^28^, which together with the observed large chemical shift changes (Fig. 4C) indicate that atRA binds to Crabp1 relatively strongly (K_D_ < 1 µM) within the Crabp1 β-sandwich as previously reported^29,30^. Figure 4C plots maximal chemical shift changes vs. the Crabp1 amino acid sequence and indicates where atRA binds to Crabp1. The most perturbed residues indicate that atRA binds within the Crabp1 β-sandwich, as previously reported^26,31^. We also note that several resonances that interact with atRA are not observed in apo-Crabp1, and that these resonances become well defined upon atRA binding. Rizo *et al*.^32^ previously proposed that residues around the atRA binding site in apo-Crabp1 are dynamic and not observed due to exchange broadening.

**Figure 5:**
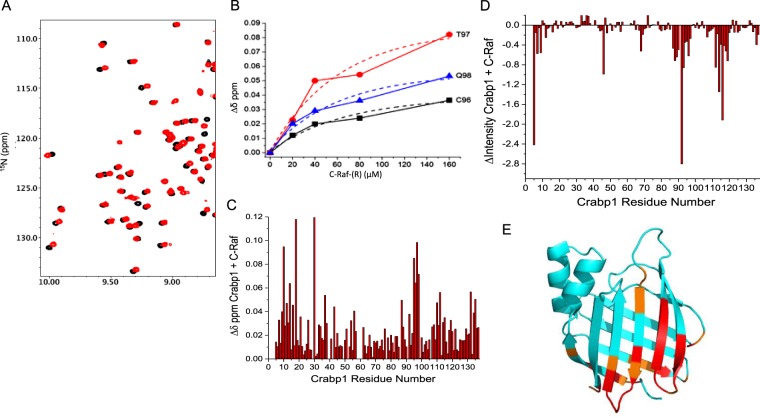
NMR analyses showing Crabp1 direct interaction with the RBD of cRaf. (**A**) An expanded region of ^1^H,^15^N HSQC spectra of the 19 µM Crabp1 in the absence (black peaks) and presence (red peaks) of 126 µM cRaf-(R) (aa 51–187). (**B**) Titration curves of selected Crabp1 residues (C96, T97, Q98) in which the chemical shift change is plotted vs. the cRaf-(R) concentration. (**C**) Maximal chemical shift changes (∆δ) induced by cRaf-(R) on Crabp1. (**D**) Maximal resonance intensity changes (∆Intensity) induced by cRaf-(R) on Crabp1. (**E**) Cartoon representation of cRaf-(R) interaction sites with marked spectral changes on Crabp1 in the presence of cRaf-(R): 1 SD – orange, 2 SD – red. PDB: 1CBR. Chemical shifts were internally referenced to DSS (4,4-dimethyl-4-silapentane-1-sulfonic acid), and chemical shift differences in panels (C,D) were calculated as [(∆δ ^1^H)^2^ + (0.25 ∆δ ^15^N)^2^]^1/2^. NMR acquisition and sample conditions are described in the “Material and Methods”.

More importantly, our NMR data demonstrate that cRaf RBD interacts with Crabp1 in solution. Figure 5A overlays ^15^N-^1^H HSQC spectral expansions of ^15^N-labeled Crabp1 (19 µM) in the absence (black peaks) and presence (red peaks) of cRaf at 126 µM. The observation that Crabp1 resonances are significantly shifted upon addition of cRaf indicates that cRaf indeed binds to Crabp1. The same effects are essentially observed in the presence of atRA. In fact, as a function of c-Raf concentration, Crabp1 chemical shift changes of three of the most shifted atRA-bound Crabp1 resonances allow us to roughly estimate a K_D_ ~ 40 µM (Fig. 5B). Figure 5C,D plot chemical shift (∆δ) and resonance intensity (∆Intensity) changes, respectively, vs. the Crabp1 amino acid sequence. Overall, the most perturbed residues are color highlighted (red for (∆δ > 2 SD) from the average; orange for (∆δ between 1 SD and 2 SD), and cyan for (∆δ < 1 SD) on a crystal structure of Crabp1 (PDB 1CBR, Fig. 5E). This analysis indicates that the most likely region for cRaf interactions is within the 6-strand β-sheet face of Crabp1. Although some effects are noted on the opposing face of the β-sandwich, these effects are likely to be indirect. This in turn suggests that Raf binding may allosterically affect Crabp1 structure/dynamics throughout other parts of Crabp1.

### Crabp1-selective compound C3 dampens growth factor-stimulated Raf activation

**Figure 6:**
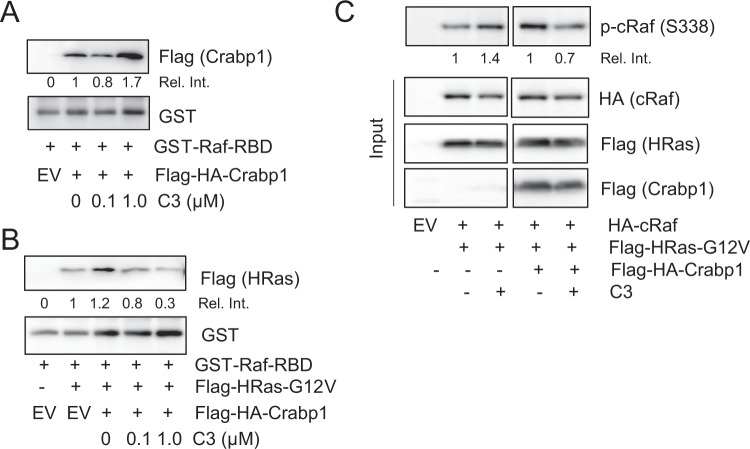
Crabp1-selective compound C3 enhances Crabp1 interaction with the Raf-RBD and competition with active Ras for RBD binding. (**A**) *In vitro* interaction assay. Compound C3 increases Crabp1 interaction with the Raf-RBD. (**B**) Compound C3/Crabp1 reduces active Ras interaction with the Raf-RBD. (**C**) HEK293T cells reconstituted with the indicated expression vectors and treated with, or without, C3 for 30 min were monitored for Raf activation in cells, indicated with p-cRaf (S338).

atRA is not an ideal therapeutic agent because it binds to and acts on a wide spectrum of biological molecules including all RARs. In a biological screening for Crabp1-selective, RAR-independent atRA-analogs, we have previously identified compound 3 (C3) with a chemical formula of C_17_H_16_NO_3_Cl, which can bind Crabp1 to activate Erk1/2 phosphorylation without eliciting genomic (RAR-mediated) effects^9^. We thus examined whether C3 could enhance Crabp1 competition with active Ras using *in vitro* direct interaction assays. Figure 6A shows that C3 indeed increases Crabp1 binding to the RBD of Raf dose-dependently. Further competition assay using oncogenic HRas-G12V mutant shows that C3-Crabp1 competes with the activated Ras also in a C3 dose-dependent manner (Fig. 6B). To further validate this mechanism in a cellular context, we used HEK293T cell clones reconstituted with cRaf and the activated Hras (G12V) in the absence of growth factors (Fig. 6C). The data show that C3 indeed dampens cRaf phosphorylation at S338 only in the presence of Crabp1 (top, right two lanes), confirming that C3, like atRA, also effectively dampens Raf-Mek-Erk activation via Crabp1 in cells. However, C3 at higher concentration (5 µM) seems to exhibit cytotoxicity in cells (data not shown).

## Discussion

The classical/canonical MAPK signaling begins with receptor tyrosine kinase activation on the cell membrane and propagates through Ras-Raf-Mek-Erk for various biological processes such as cell proliferation, cell cycle regulation, cell survival and apoptosis, etc. In a cancer context, the MAPK pathway has a well-documented role in contributing to disease progression and metastasis. Therefore, multiple components of this signaling pathway provide attractive therapeutic targets for cancers^11^. However, current attempts to target MAPK components have yielded little success, highlighting a need for novel therapeutic strategies.

We and others have shown that atRA, a primarily differentiating agent that is known to act via its nuclear receptors (RARs), can also crosstalk with this MAPK signaling to rapidly stimulate or suppress Erk phosphorylation in various cellular contexts^9,33–35^. We have shown, in the physiologically relevant system of ESC culture, that atRA modulates Erk activation in a bi-phasic manner. First is a rapid activation (within minutes), which is followed by a delayed activation (after 6–12 hrs)^8^. The first rapid activation of Erk by atRA was RAR-independent, which led us to propose a non-canonical atRA signaling pathway now recognized to be mediated by Crabp1. Importantly, this newly established Crabp1-dependent signaling pathway of atRA can modulate cell cycle of ESC in cultures and NSC pool expansion in the hippocampus^8,10^. These results first revealed a previously unrecognized physiological function of Crabp1. We recently found that Crabp1 can also act as a signaling molecule to modulate calcium/calmodulin dependent protein kinase II in a mouse heart failure model where Crabp1 functions to protect against heart failure in the adult mice^36^. It is increasingly clear that Crabp1-mediated signaling is physiologically relevant and significant. This current study demonstrates, for the first time, how Crabp1 functions at the molecular level, further supporting the biological significance of this non-canonical, RAR-independent, activity of atRA. With respect to the regulation of growth factor-stimulated MAPK signaling, it is most exciting to find that atRA-Crabp1 directly targets Raf, given Raf kinase is a highly desirable therapeutic target in diseases such as cancer^37^.

**Figure 7:**
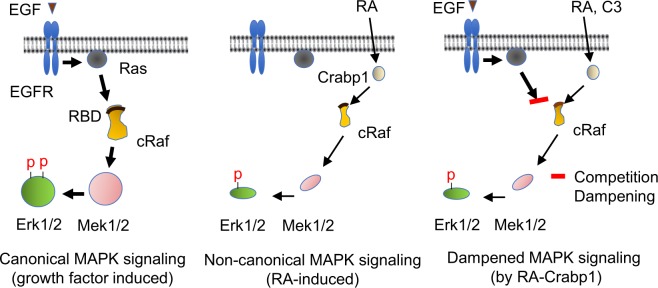
A model of atRA-Crabp1 elicited non-canonical activity. Left: Growth factors such as EGF induce strong canonical MAPK signaling via Ras binding to Raf to activate Raf kinase, which subsequently phosphorylates Mek1/2 and then Erk1/2. Middle: atRA, upon binding to Crabp1, non-canonically activates Raf kinase (in the absence of EGF) which weakly activates Mek1/2 and Erk1/2. Right: In cells that are simultaneously exposed to EGF and atRA, atRA-Crabp1 competes with active Ras for Raf interaction, thereby dampening EGF/EGFR-Ras signaling and rendering only weak phosphorylation of Erk1/2 in spite of EGF stimulation.

In a physiological context, two potential effects of atRA/Crabp1 on MAPK signaling occur, either weak activation of Erk1/2 in the absence of EGF or dampening EGF-stimulated Erk1/2 activation (model in Fig. 7). The contextual dependency of holo-Crabp1 functions is consistent with most of the previous observations that atRA can either activate or suppress Erk activity, depending on cell types and culture conditions^8,9,38–40^. This also suggests a need for careful evaluation of future studies conducted in different types of cells and/or in various culture conditions.

This non-canonical signaling pathway of atRA in conjunction with MAPK pathway also suggests a novel paradigm for signal crosstalk between the endocrine and growth factor axes (Fig. 7). Proliferating cells (such as normal stem cells) may be exposed to different growth factors for continuous proliferation and/or differentiating signals such as atRA that canonically activates nuclear programs to alter gene expression for cellular differentiation. Then cells can either proliferate normally (when atRA is absent or low and growth factors are available) or prepare for differentiation (when atRA concentration is elevated and/or growth factors are withdrawn). In this latter situation (atRA present, prime for differentiation), cell proliferation must first decrease. This decrease could be facilitated by the initial non-genomic action of atRA to dampen growth factor sensitivity. Indeed, our previous study has found that this atRA-Crabp1 mediated Erk signaling slowed down cell cycle progression^8^. This will better prepare cells for differentiation typically mediated by the delayed RAR-dependent genomic action of atRA. Over all, the Crabp1-mediated crosstalk may represent a delicate cellular program required for coordinating the nuclear and extra-nuclear environments when an important decision, such as proliferation vs. differentiation, has to be made.

From a therapeutic point of view, retinoids, especially atRA, have attracted initial enthusiasm because of the ability to trigger cancer cell differentiation or apoptosis^41,42^. However, this therapy exhibited a wide spectrum of toxicity (retinoid toxicity) caused by RARs^43^. The newly identified non-canonical action of atRA, mediated by Crabp1 but not RARs, sheds a new light on an alternative strategy of retinoid therapy. By using Crabp1-selective compounds, it is possible to dampen growth signal in certain cancers without eliciting RAR-mediated retinoid toxicity. In this current study, we indeed have utilized such a compound, C3, to demonstrate a proof-of-concept for this strategy in cell cultures. Further extended studies are needed to validate this strategy.

It is known that Ras, a GTPase, can be activated by RTK activity of EGFR or mutations such as G12V that result in constitutive activity. Ras then binds to the RBD of Raf, which then undergoes a conformational change to adopt the active open structure of Raf kinase domain^12,16^. In this current study, we first focused on the RBD-interaction surface of Crabp1. NMR HSQC analyses reveal that the 6-strand β-sheet surface area of Crabp1 makes direct contact with the RBD. To understand detailed molecular features underlying the competition between Crabp1 and active Ras with regards to Raf regulation, more extensive molecular and structural studies are needed. For instance, it will be important to evaluate conformational changes on the surface of Raf as Crabp1 binds to its RBD, in comparison to the binding of active Ras.

This study provides evidence for a novel regulatory mechanism to modulate Raf kinase activation by the action of an endocrine factor, atRA, together with its cytosolic binding protein, Crabp1. Although phosphorylation on S338 (BRaf equivalent S445) is a hallmark of Raf activation, the upstream kinase that targets S338 remains elusive. Studies implicating Pak1, Pak3, Ck2, and other kinases in the upstream signaling of Raf have been observed. But whether or not the activity of these kinases is essential for Raf activation remains controversial^44–47^. In addition, studies have indicated that Raf itself as a homodimer or heterodimers with other Raf isoforms can also alter S338 phosphorylation^48^. These previous studies underscore the need to further investigate the regulation of Raf activity in various cellular environments or growth conditions.

In this current study we report that Crabp1 may act as a scaffold, or an allosteric regulator, that forms a atRA-inducible complex with at least the MAPK cascade components- RAF, MEK, ERK (Fig. 1C). Interestingly, Crabp1 appears to compete with KSR1, the established scaffold that mediates growth-factor mediated MAPK activation (Fig. 1G) (Kolch 2005). This would suggest that Crabp1 may act by competing with KSR1 for Raf binding, modulating phosphorylation at S338 via the above-mentioned kinases, other Raf molecules, or some unknown upstream kinases. We thus propose a new endocrine (atRA)-elicited mechanism that can also regulate the MAPK kinase pathway and that cross-talks with the canonical growth factor-stimulated MAPK pathway (Fig. 7). Further study is needed to elucidate the particular upstream events that result in Raf activation upon atRA-Crabp1 binding. Nevertheless, our study highlights the increasing complexity of Raf activation mechanisms. Further studies are needed to compare and contrast classical growth-factor activated mechanisms and this novel endocrine (atRA) elicited mechanisms with regards to the regulation of MAPK signaling pathway.

## Materials and Methods

### Cell Culture and plasmid constructs and transfection

Experimental procedures were conducted according to NIH guidelines and approved by the University of Minnesota Institutional Animal Care and Use Committee. CJ7 (WT) ESCs and CKO ESCs derived from 2.5 day-morula of CKO mice^9^ were maintained as described^49,50^. Briefly, cells were maintained in DMEM medium supplemented with 17% ESC-qualified fetal bovine serum, 2 mM glutamine, 0.1 mM nonessential amino acids, 6 μM β-mercaptoethanol, 2 mM HEPES, and 1,000 U/ml recombinant leukemia inhibitory factor (LIF2005, Sigma-Aldrich, St. Louis, MO). Cells were grown on irradiated mouse embryonic feeder cells in 0.2% (w/v) gelatin-coated plates. Medium was renewed every day. Cells (passage number less than 10) were treated with 100 nM atRA (R2625, Sigma-Aldrich, St. Louis, MO). HEK293T cells were maintained in high glucose DMEM supplemented with 10% (v/v) FBS and 1% (v/v) Penicillin/Streptomycin.

Mouse *Crabp1* (NM_013496.3) and *Hras* (NM_001130443.1) cDNA were cloned into Flag tagged pCMX-PL1, and mouse *Braf* (NM_139294.5) and *Raf1* (NM_001356333.2) were cloned into HA-tagged pCMX-PL1. Mutation (G12V) in *Hras* was introduced with site-directed mutagenesis kit (Agilent Technologies, Cedar Creek, TX). His-Crabp1 was cloned into pET-15b (69665, Millipore Sigma) to generate N-terminal, His6x-tag. For His-cRaf-(R) fragment, *Raf1* aa 51–187 was cloned into a pET-32a vector (69015, Millipore Sigma). Flag-HA-Crabp1 expression lentivirus construct was cloned into pCDH-EF1α-MCS-IRES-puro (System Biosciences) and prepared as described^51^. Stable clones expressing Flag-HA-Crabp1 and empty vector were established in HEK293T cells using puromycin selection and transfected by routine calcium phosphate method for the reconstitution of MAPK signaling components.

### *In situ* proximity ligation assay

Cells were fixed by 4% (w/v) paraformaldehyde for 10 minutes and permeabilized using 0.2% (v/v) Triton X-100 for 5 minutes at 4 °C. *In situ* proximity ligation assay was performed using Duolink *in situ* PLA detection kit (DUO94004, Sigma-Aldrich, St. Louis, MO) according to the protocol provided by the manufacturer. Images were acquired by Olympus FluoView FV1000 BX2 upright confocal microscope at 40x magnification. Antibodies used to detect endogenous complex are against Crabp1 (c1608, Sigma-Aldrich), cRaf (#53745, D4B3J, Cell Signaling, Danvers, MA), Mek1/2 (sc-436, Sant Cruz Biotechnology, Santa Cruz, CA), and p-Erk1/2 (#9101, Cell Signaling). The red fluorescent punctate represents protein complex and images acquired from different fields of individual experiment was counted with ImageJ image processing freeware.

### *In vitro* kinase assay

*Semi in vitro* kinase assay was performed as described^36^. T7 coupled HA-cRaf was synthesized with TnT® Quick Coupled Transcription/Translation System (Promega, Madison, WI). Recombinant unphosphorylated Mek1 (0.4 µg) (14–429, Millipore-Sigma) was incubated with TnT HA-cRaf, 1 µg purified His-Crabp1 in the total volume of 35 µl kinase buffer (20 mM MOPS, pH 7.2, 0.1 mM ATP, 25 mM β-glycerophosphate, 5 mM EGTA, 1 mM sodium orthovanadate, 1 mM DTT, 18 mM MgCl_2_, and 1x protein inhibitor cocktail) with and without 100 nM atRA at 30 °C for 15 min. At the end of the reaction, SDS loading buffer was added and boiled. Mek1 phosphorylation at T286 was detected by western blot.

### *In vitro* pulldown assay and co-immunoprecipitation

TnT HA-tagged BRaf and cRaf were incubated with 4.25 µg purified His-Crabp1 and atRA for 30 min in 0.5 ml co-immunoprecipitation (Co-IP) buffer (50 mM Tris-HCl, pH 8.0, 150 mM NaCl, 0.2% (v/v) NP-40, 10% (v/v) glycerol, and 1 mM EDTA) and pulled down with G agarose beads (16–266, Sigma-Aldrich) and anti-HA antibody or normal rabbit IgG at 4 °C overnight. After washing, beads were subjected to western blot. For the RBD interaction assay, His-cRaf-(R) and His-tagged empty plasmid pET-32a (EV) were induced in Rosetta^TM^ DE3 *E. Coli* with 1 mM Isopropyl β-D-1-thiogalactopyranoside (IPTG). Induced proteins were captured with Ni-NTA agarose beads (Qiagen Sciences, MD) in the presence of 20 mM imidazole. For interaction and competition assay, we used His-cRaf-(R) or GST-Raf-RBD (Cytoskeleton Inc.) or GST-Raf1-RBD (Millipore Sigma (14–863) beads to pull down Flag-HA-Crabp1 expressed in HEK293T cells and TnT Flag-Ras (WT or G12V) following incubation with atRA as above in 0.8 ml of modified Co-IP buffer (50 mM Tris-HCl, pH 8.0, 150 mM NaCl, 10 mM MgCl_2_, 0.2% (v/v) NP-40, and 10% (v/v) glycerol). For Co-IP experiments, 293T cells were transfected with Flag-HA-Crabp1 only or both myc-cRaf and Flag-HA-Crabp1 and treated with 100 nM atRA for 15 min. Cell lysates were precipitated with either M2 beads or c-Myc antibody followed by western blot analysis.

### Western blot analysis

Whole cell lysates were prepared with RIPA buffer (25 mM Tris-HCl, pH 7.4, 150 mM NaCl, 1% (v/v) NP-40, 1% (w/v) Na-deoxycholate and 1% (w/v) Na-dodecyl sulfate) as described. Antibodies against β-actin (SC-47778), Erk1 (SC-93), and Erk2 (SC-153), His-probe (sc-8036), Ksr1 (SC-515924), cMyc (SC-56634), and HA (sc-7392) were from Santa Cruz Biotechnology. Antibodies against Crabp1 (C1608) and Flag (F3165) were from Millipore Sigma. Antibodies against Mek1 (#12671), p-Mek1 (#9127), Mek2 (#9147), p-Erk1/2 (#9101), p-cRaf S259 (#9421), and p-cRaf S338 (#9427) were from Cell Signaling. AtRA and hEGF (SRP3027) were from Sigma-Aldrich. Samples were separated on 10% or 13.8% (v/v) SDS polyacrylamide gel, transferred to PVDF membrane, which were cut and probed and re-probed with antibodies to detect protein expression levels, and developed by myECL Imager (Thermo Scientific).

### Protein expression and purification

For native Crabp1, Rosetta^TM^ DE3 *E. coli* containing the pET-15b Crabp1 construct was grown in LB + ampicillin (150 µg/mL) at 37 °C, 250 rpm until OD_600_ ~0.6–0.8. Protein expression was then induced with 1 mM IPTG at 25 °C, 250 rpm overnight. For ^15^N and ^13^C Labeled Crabp1, a single colony from the plate was used to inoculate a 3 ml starter culture in LB media + ampicillin (150 µg/mL) and grown at 37 °C, 250 rpm to OD_600_ ~0.4–0.6. The starter culture was transferred to a 125 ml sterile Erlenmeyer flask containing 25 ml of M9 media + ampicillin (150 µg/ml), containing^15^NH_4_Cl and ^13^C-glucose as the sole source of nitrogen and carbon.^15^N Isogro (606871, Sigma-Aldrich) was also supplemented along with 0.5 mg amount of biotin and thiamine. When the OD_600_ reached ~0.4–0.6, the starter culture was transferred to the fernbach flask containing 1 liter of the same M9 media recipe (see above). Protein was induced with 1 mM IPTG when OD_600_ reached ~0.8–0.9 for 4 hours at 37 °C, 250 rpm. Uniformly ^15^N labeled Crabp1 was expressed as described without addition of ^12^C-glucose for titration experiments. Native Crapb1, uniformly ^15^N, and uniformly ^15^N &^13^C labeled His-tagged Crabp1 was affinity-captured on Ni-NTA beads (Qiagen) and purified in 30 mM Tris-acetate, pH 6.2, 75 mM Na_2_SO_4_, 10 μM ZnCl_2_, 1 mM DTT. Concentrated protein was concentrated in NMR buffer (30 mM d_11_-Tris-d_3_-acetate, pH 6.2, 75 mM Na_2_SO_4_, 10 μM ZnCl_2,_ 1 mM TCEP) using Amicon® Ultra-0.5 Centrifugal Filters (UFC5010, Sigma-Aldrich) to 2 mM or 100 µM. For c-Raf-(R) fragment (51–187), His-tagged c-Raf-(R) plasmid was transformed into Rosetta^TM^ DE3 *E. coli* host strain. His-c-Raf fragment (aa 51–187) was purified unlabeled as described above and concentrated to 400 µM. Purified proteins were confirmed by Coomassie blue staining and western blot.

### NMR spectroscopy

NMR experiments were performed at 25 °C on Bruker Avance III 700, 900-MHz and Avance Neo 600-MHz spectrometer, each equipped with a 5 mM TCI cryoprobe. Spectra were processed using NMRPipe^52^ and analyzed with Sparky^53^ and NMRViewJ^54^. Sequence-specific backbone assignments were completed using two three-dimensional spectra, HN(CO)CACB, HNCACB. Peaks were fully assigned using the NMRFAM-Sparky package^53,55^ referencing BMRB ID# 19271^32,56^. For titration experiments, uniformly ^15^N-labeled Crabp1 was dissolved at a concentration of 19 μM in 30 mM d_11_-Tris-d_3_-acetate, pH 6.2, 75 mM Na_2_SO_4_, 10 µM ZnCl_2_, 1 mM DTT, made up using a 95% H_2_O/5% D_2_O mixture (v/v). ^1^H-^15^N Heteronuclear single quantum coherence (HSQC) NMR spectra were acquired for ^15^N Crabp1 and increasing amounts of unlabeled His-c-Raf-(R) aa 51–197, in the presence and absence of RA (40 μM). Chemical shift differences were calculated as [(∆^1^H)^2^ + (0.15∆^15^N)^2^]^1/2^. Intensity changes (∆Int) were calculated as (1-Int_i_/Int_0_). Additional experiments were performed using 19 µM ^15^N-labeled Crabp1 +/− 21 µM RA +/− 126 µM c-Raf-(R).

### Statistical analysis

Band intensities were determined by densitometric scanning using ImageJ, and relative fold intensities are numerically marked under each relevant panel. Comparisons between groups were evaluated with Student’s t-test. Differences were considered significant at P < 0.05. All values are presented as mean ± SEM.

## Supplementary information

Supplementary Information
