# Publisher Correction: A new regulatory mechanism for Raf kinase activation, retinoic acid-bound Crabp1

**DOI:** 10.1038/s41598-019-53692-3

**Published:** 2019-11-14

**Authors:** Sung Wook Park, Jennifer Nhieu, Shawna D. Persaud, Michelle C. Miller, Youlin Xia, Yi-Wei Lin, Yu-Lung Lin, Hiroyuki Kagechika, Kevin H. Mayo, Li-Na Wei

**Affiliations:** 1 Department of Pharmacology, University of Minnesota, Minneapolis, MN 55455 USA; 2 Department of Biochemistry, Molecular Biology & Biophysics, University of Minnesota, Minneapolis, MN 55455 USA; 3 Minnesota NMR Center, University of Minnesota, Twin Cities, Minneapolis, Minnesota 55455 USA; 4 Tokyo Medical and Dental University, Institute of Biomaterials and Bioengineering, Tokyo, Japan

Correction to: *Scientific Reports* 10.1038/s41598-019-47354-7, published online 29 July 2019

In the original version of this Article, the author Sung Wook Park was incorrectly indexed. This error has now been corrected.

