# Supplementary material for: A new regulatory mechanism for Raf kinase activation, retinoic acid-bound Crabp1
Source: Sci Rep. 2019 Jul 29;9:10929. doi: 10.1038/s41598-019-47354-7 (PMC6662813; doi:10.1038/s41598-019-47354-7)
Supplement: Supplementary file 1 — Supplementary Information [file 41598_2019_47354_MOESM1_ESM.pdf]

Supplementary Information

A new regulatory mechanism for Raf kinase activation, retinoic acid-bound Crabp1

Sung Wook Park[1,#], Jennifer Nhieu[1,#], Shawna D. Persaud[1,#], Michelle C. Miller[2,#], Youlin Xia[3], Yi-Wei Lin[1], Yu- Lung Lin[1], Hiroyuki Kagechika[4], Kevin H. Mayo[2], and Li-Na Wei[1,*]

1. Department of Pharmacology University of Minnesota, Minneapolis, MN 55455, USA.
2. Department of Biochemistry, Molecular Biology & Biophysics, University of Minnesota, Minneapolis, MN 55455, USA.
3. Minnesota NMR Center, University of Minnesota, Twin Cities, Minneapolis, Minnesota 55455, USA.
4. Tokyo Medical and Dental University, Institute of Biomaterials and Bioengineering, Tokyo, Japan.

# These authors contributed equally to this study.

* Correspondence
Li-Na Wei, Department of Pharmacology, University of Minnesota Medical School, Minneapolis, MN 55455. Tel: 612-6259402; email “weixx009@umn.edu”

Figure 1

For all data:

- Membranes cut according to protein size markers were probed with antibodies.
- The parts of the blots encircled with red rectangles are presented.

Fig 1C

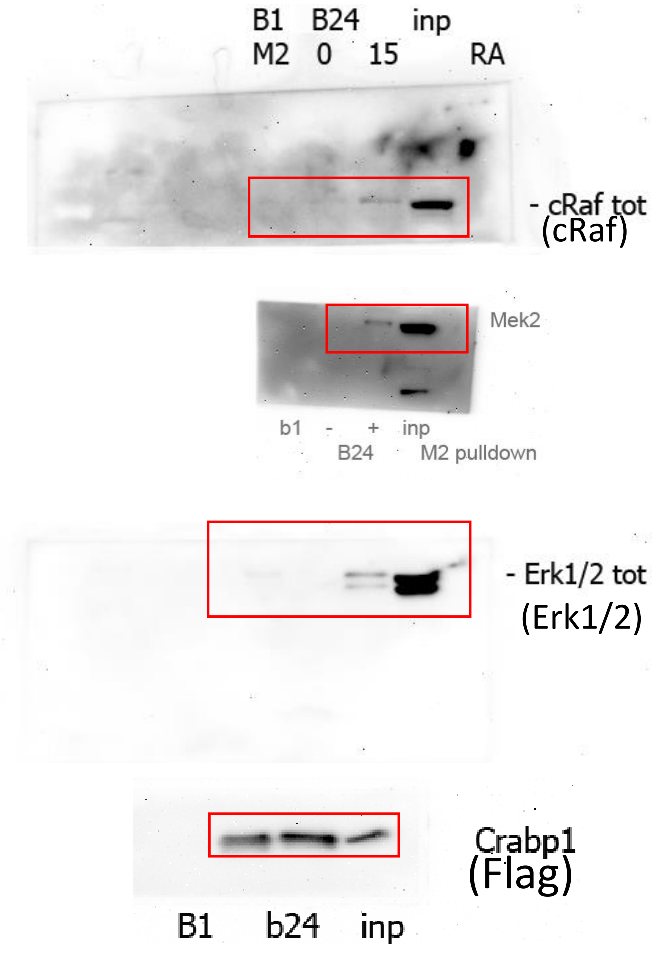

Fig 1D

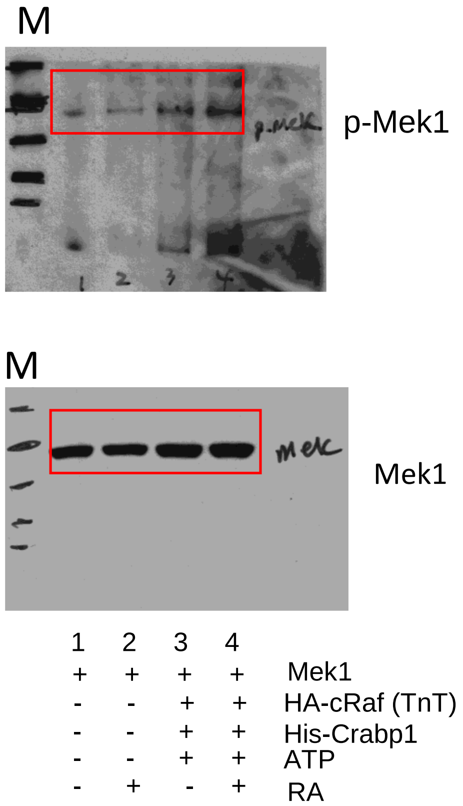

Fig 1E

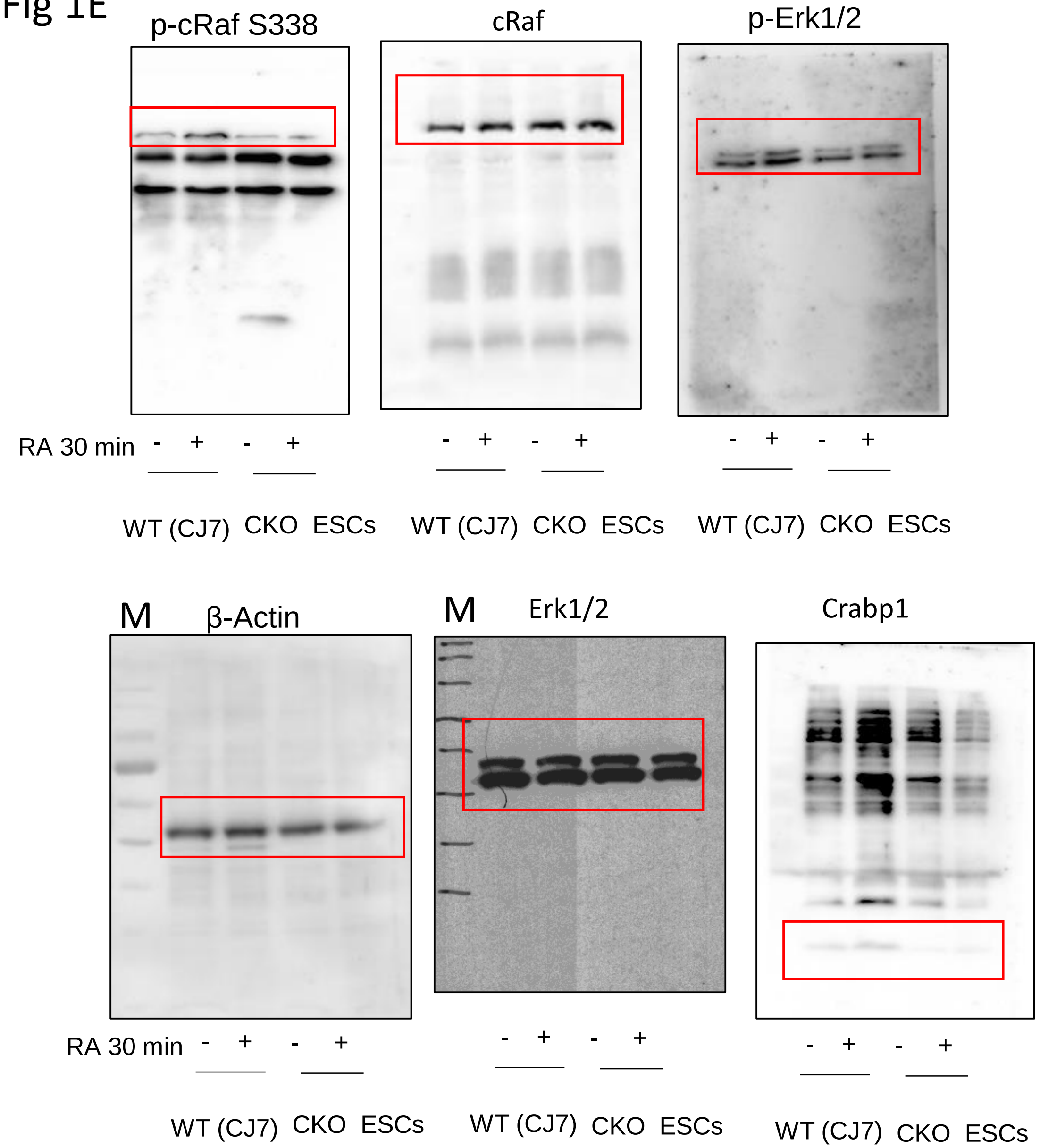

"M"- Molecular Weight Marker

Figure 1 Cont’d

Fig 1F

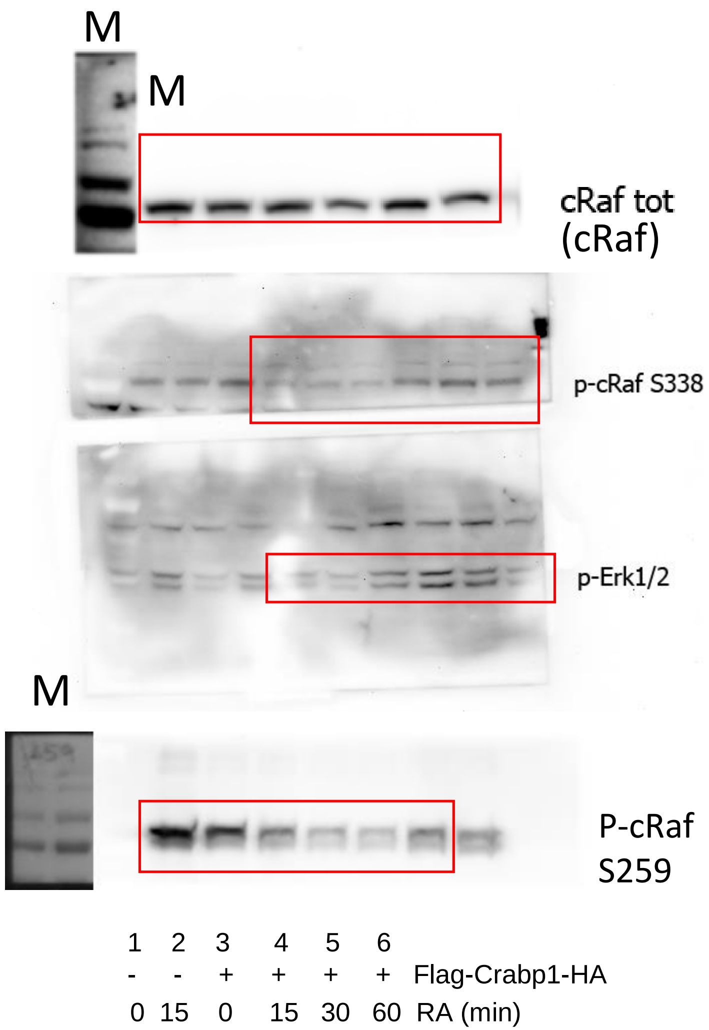

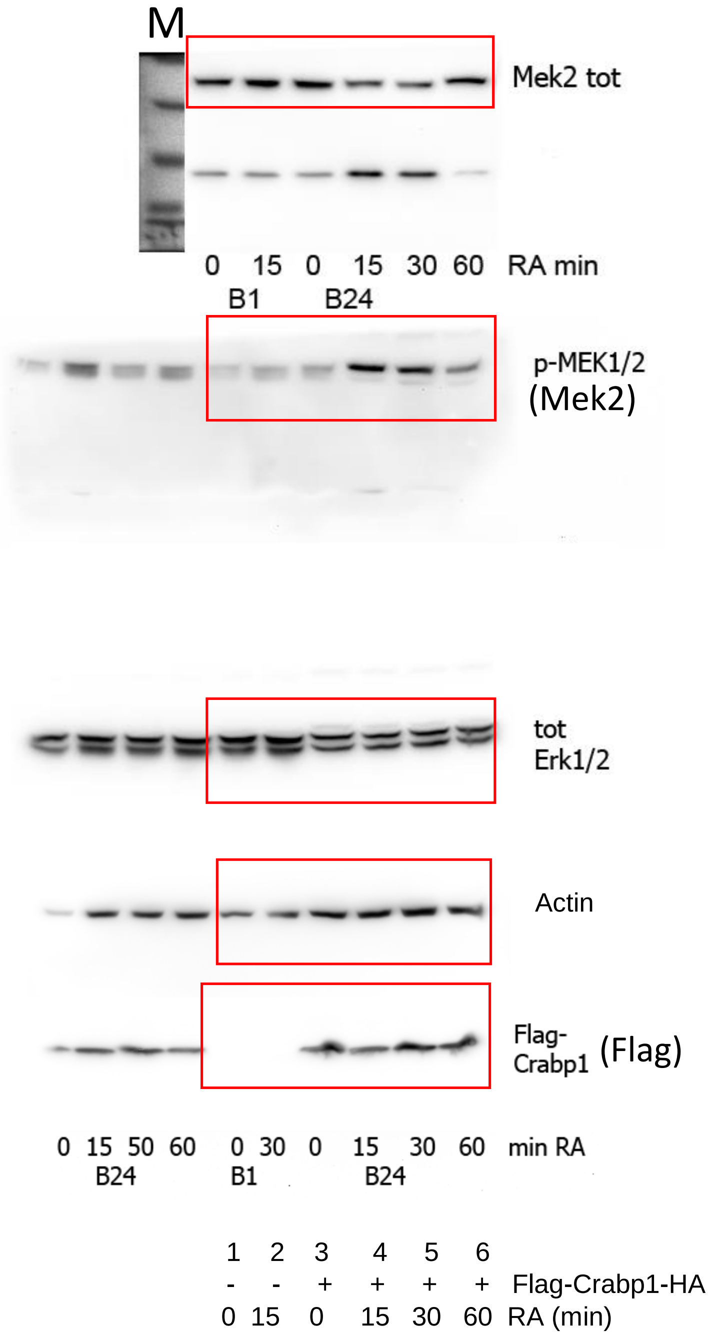

Fig 1G

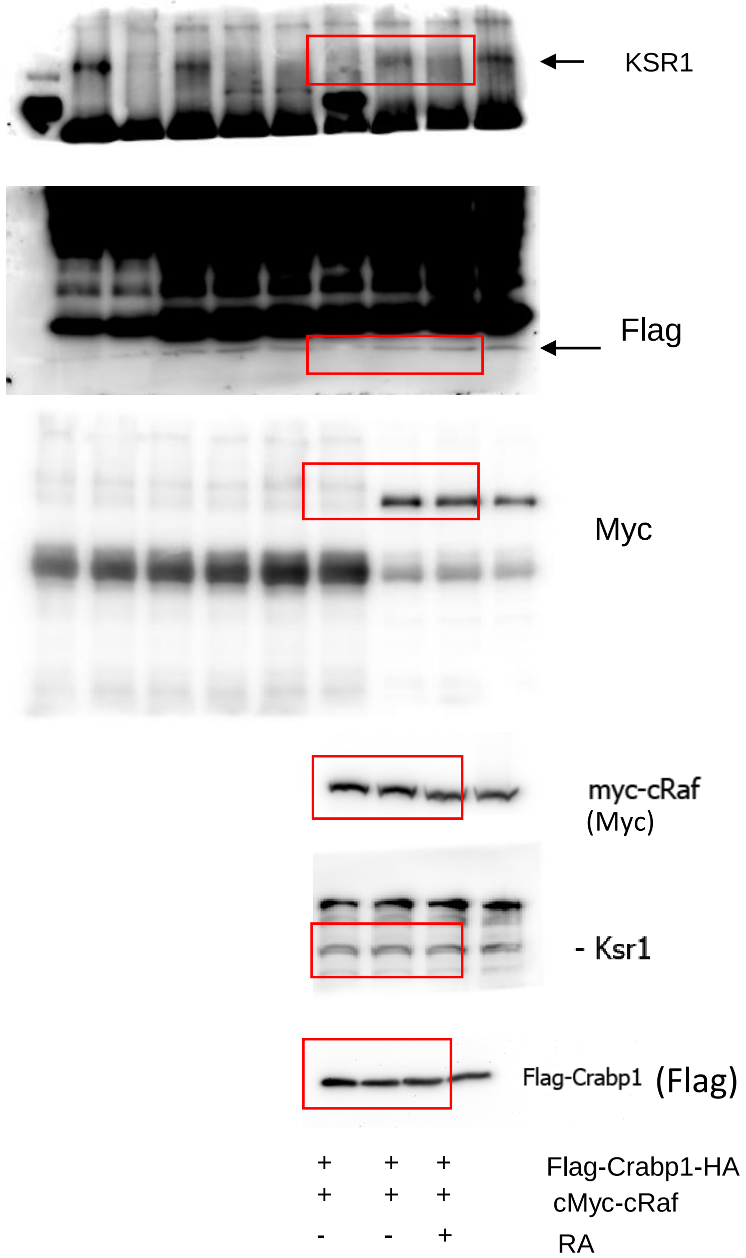

“M”- Molecular Weight Marker

Figure 2

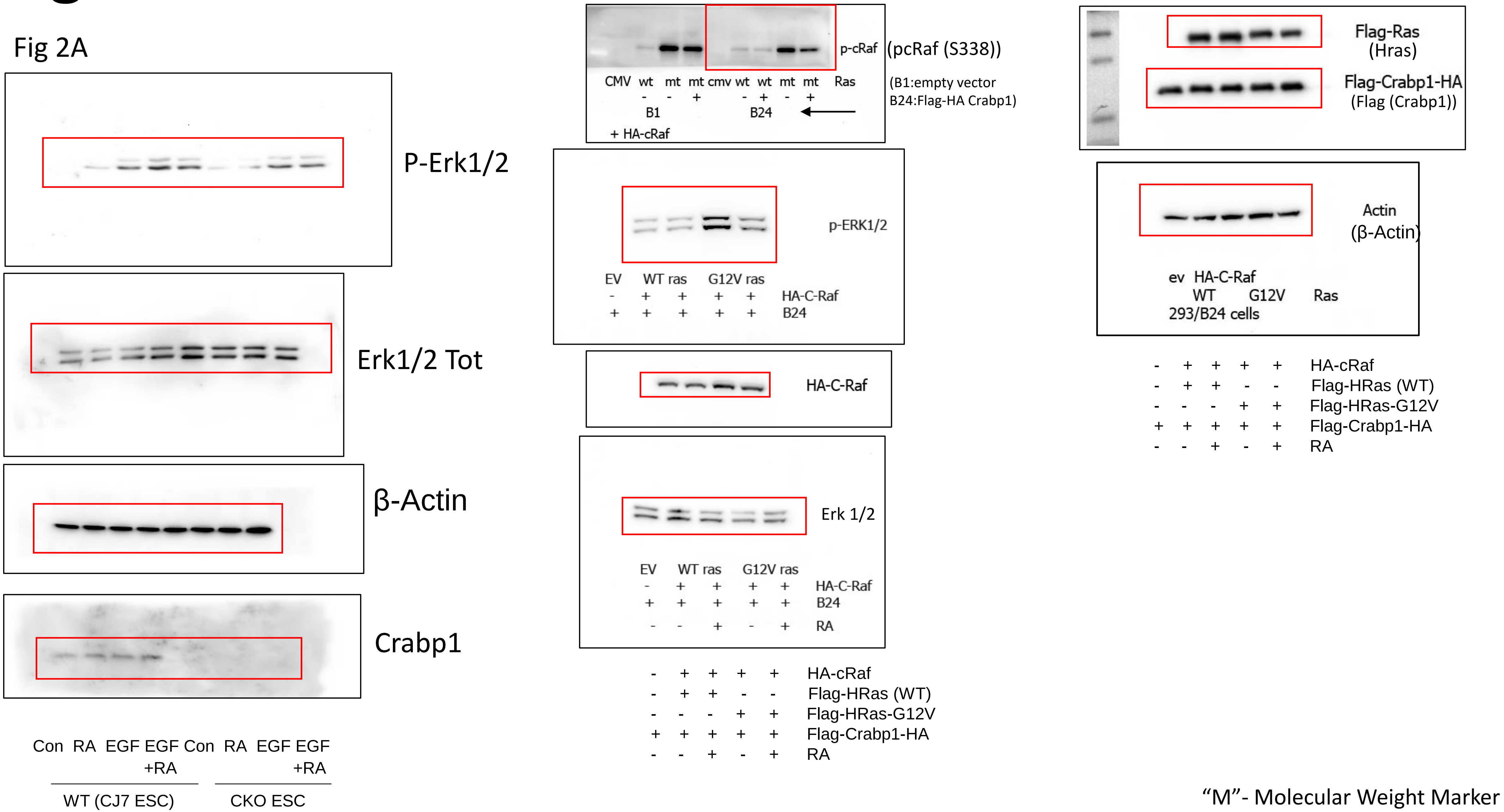
Fig 2A
P-Erk1/2
Erk1/2 Tot
β-Actin
Crabp1
Con RA EGF EGF +RA Con RA EGF EGF +RA
WT (CJ7 ESC)
CKO ESC
Fig 2B
Empty Vector Control
p-cRaf
(pcRaf (S338))
CMV wt mt mt cmv wt wt mt mt Ras
- - + - + - +
B1
B24
+ HA-cRaf
(B1:empty vector
B24:Flag-HA Crabp1)
p-ERK1/2
EV WT ras G12V ras
- + + + + HA-C-Raf
+ + + + + B24
HA-C-Raf
Erk 1/2
EV WT ras G12V ras
- + + + + HA-C-Raf
+ + + + + B24
- - + - + RA
- + + + + HA-cRaf
- + + - - Flag-HRas (WT)
- - - + + Flag-HRas-G12V
+ + + + + Flag-Crabp1-HA
- - + - + RA
Flag-Ras
(Hras)
Flag-Crabp1-HA
(Flag (Crabp1))
Actin
(β-Actin)
ev HA-C-Raf
WT G12V Ras
293/B24 cells
- + + + + HA-cRaf
- + + - - Flag-HRas (WT)
- - - + + Flag-HRas-G12V
+ + + + + Flag-Crabp1-HA
- - + - + RA
"M"- Molecular Weight Marker

Figure 2 Cont'd

Fig 2C

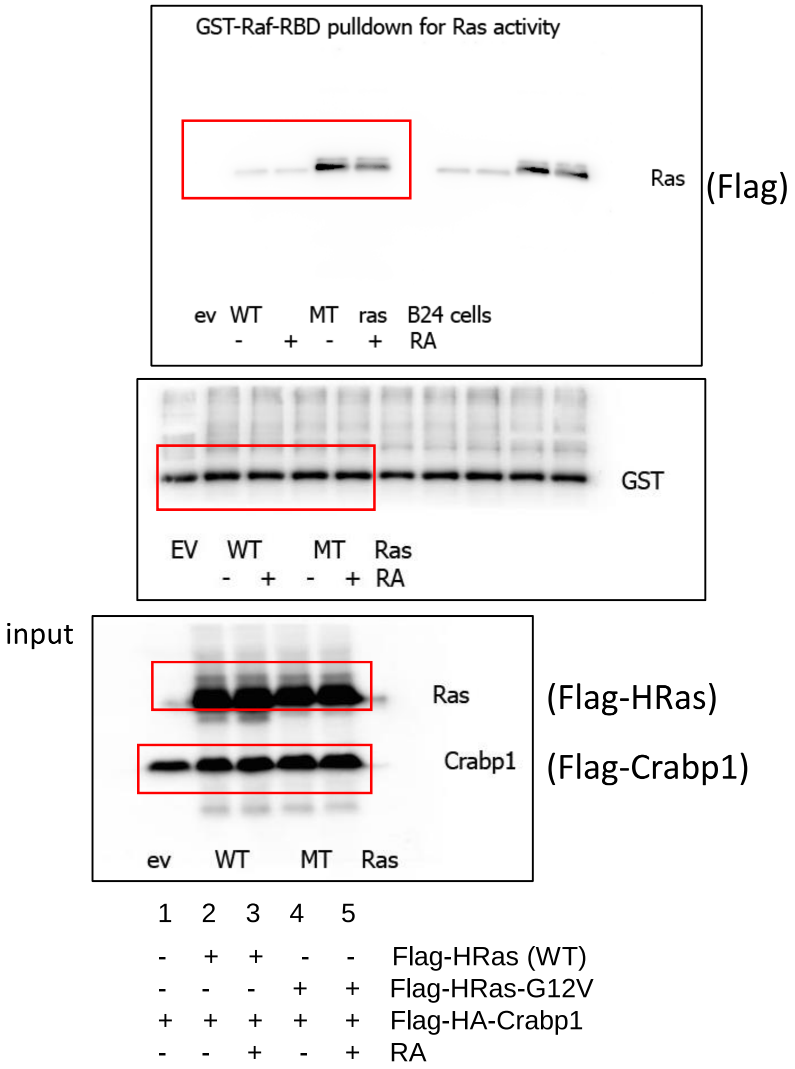

Figure 3

Fig 3A

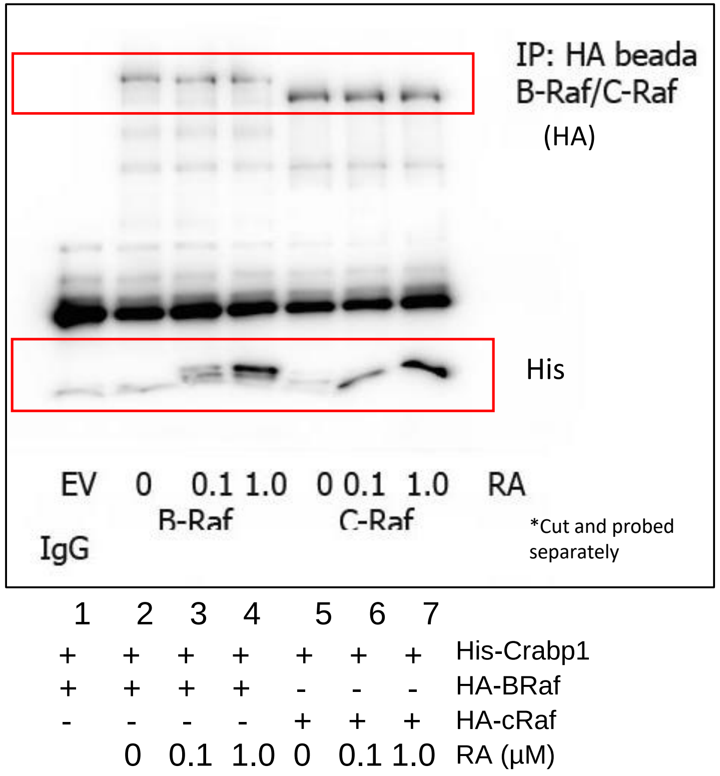

Fig 3B

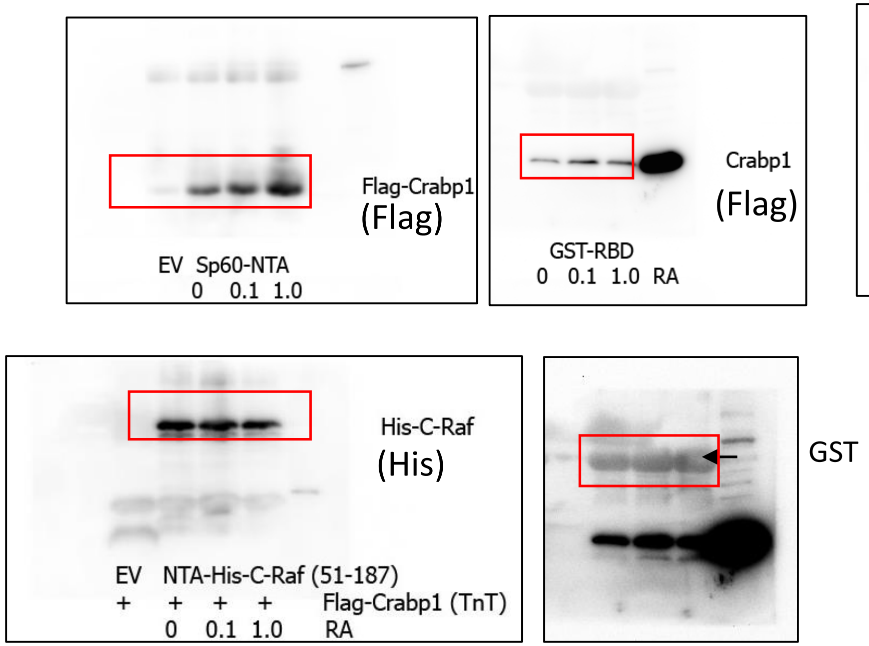

Fig 3C

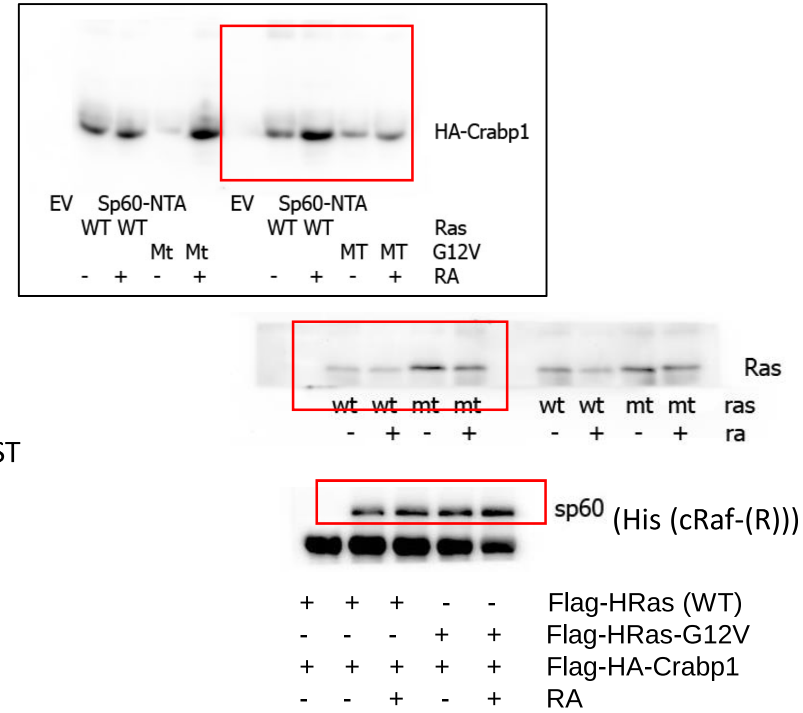

Figure 3 cont'd

Fig 3D

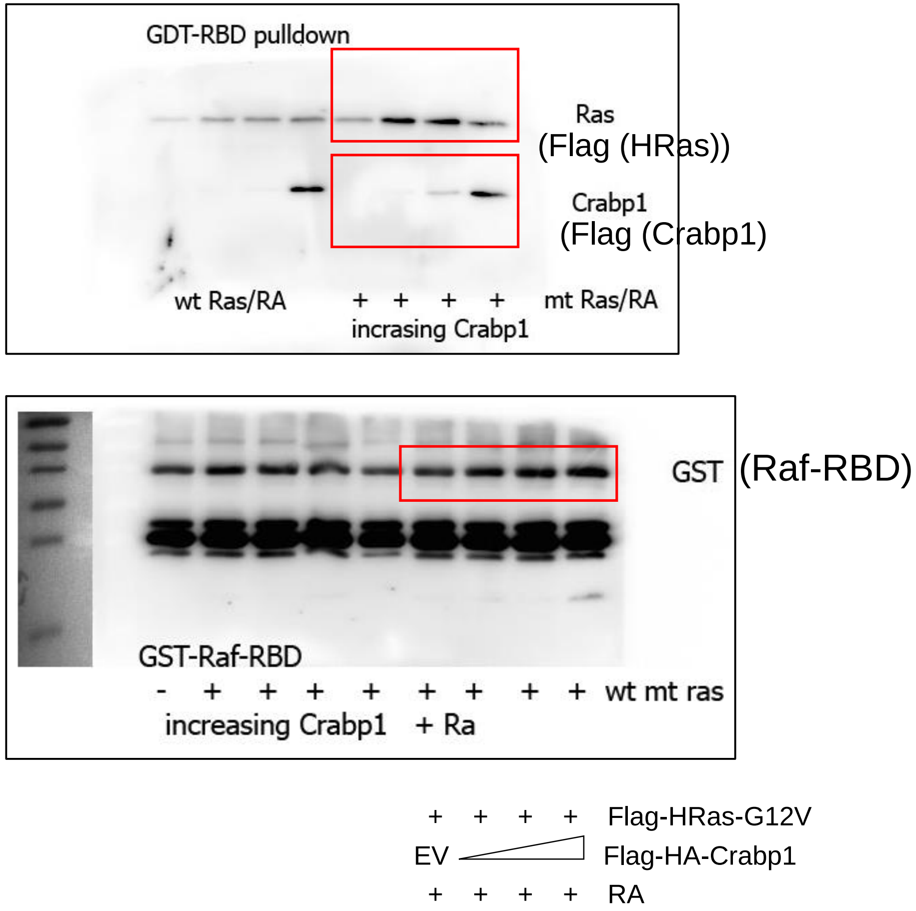

Fig 3E

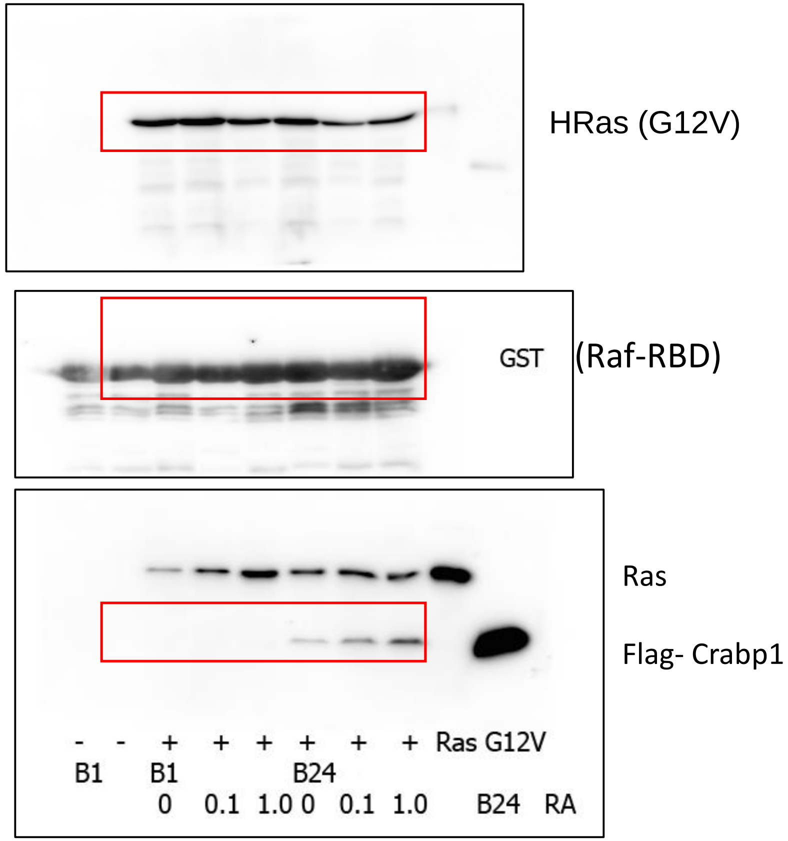

Fig 3F

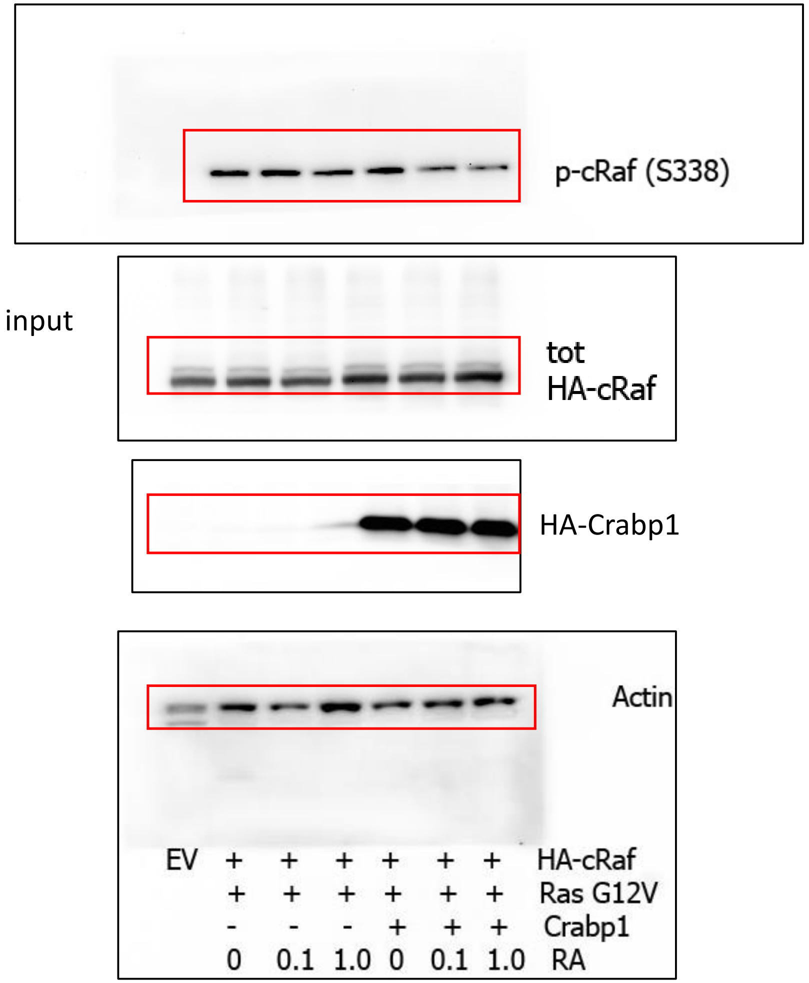

Figure 6

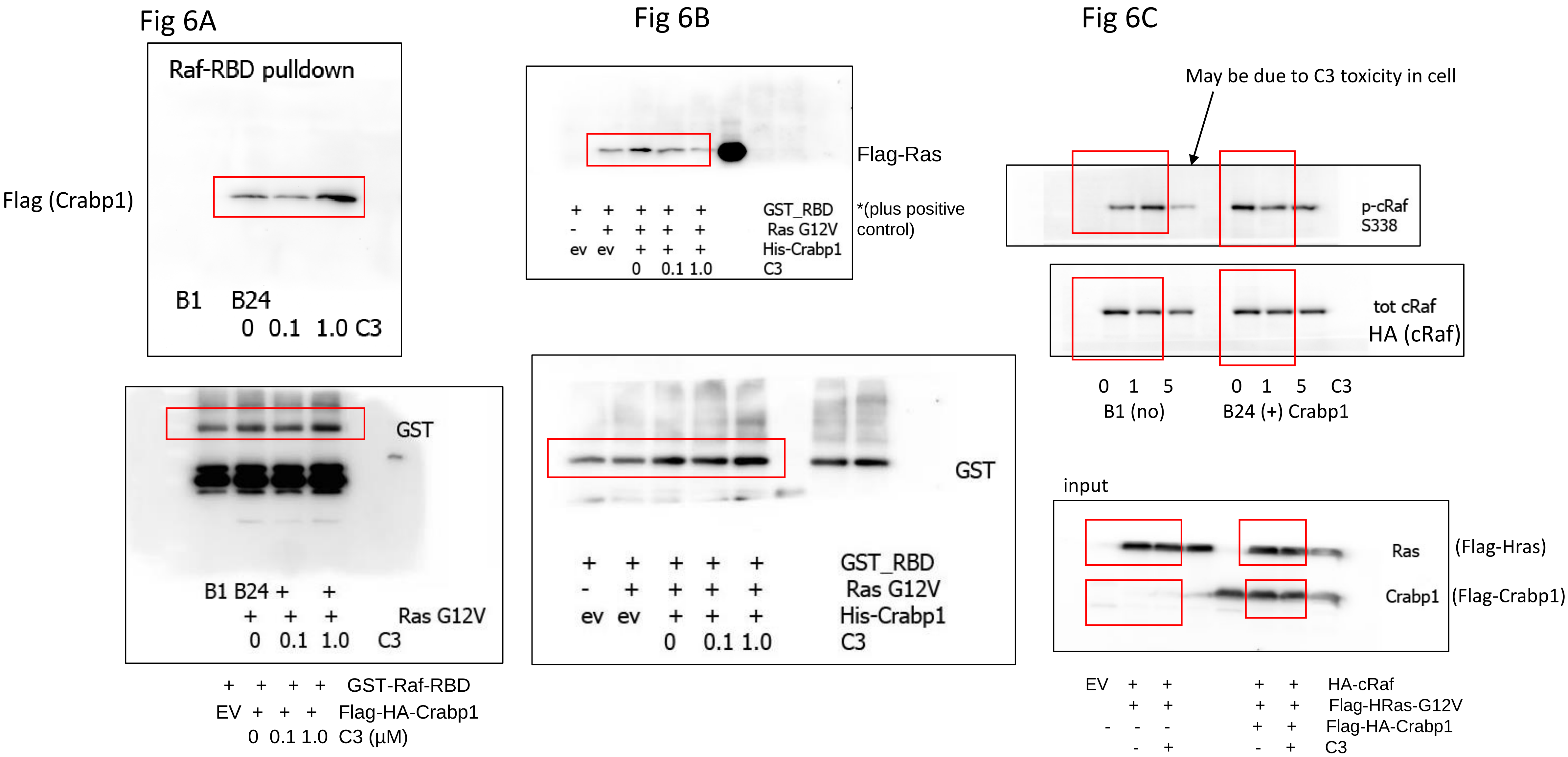

Fig 6A
Raf-RBD pulldown
Flag (Crabp1)
B1 B24
0 0.1 1.0 C3
GST
B1 B24 + +
+ + + Ras G12V
0 0.1 1.0 C3
+ + + + GST-Raf-RBD
EV + + + Flag-HA-Crabp1
0 0.1 1.0 C3 (μM)
Fig 6B
Flag-Ras
+ + + + + GST_RBD
- + + + + Ras G12V
ev ev + + + His-Crabp1
0 0.1 1.0 C3
*(plus positive control)
GST
+ + + + + GST_RBD
- + + + + Ras G12V
ev ev + + + His-Crabp1
0 0.1 1.0 C3
Fig 6C
May be due to C3 toxicity in cell
p-cRaf S338
tot cRaf
HA (cRaf)
0 1 5 0 1 5 C3
B1 (no) B24 (+) Crabp1
input
Ras (Flag-Hras)
Crabp1 (Flag-Crabp1)
EV + + + + HA-cRaf
+ + + + Flag-HRas-G12V
- - - + + Flag-HA-Crabp1
- + - + C3
